# Diagnostic performance of multi-organ ultrasound with pocket-sized device in the management of acute dyspnea

**DOI:** 10.1186/s12947-017-0105-8

**Published:** 2017-06-19

**Authors:** Alfonso Sforza, Costantino Mancusi, Maria Viviana Carlino, Agostino Buonauro, Marco Barozzi, Giuseppe Romano, Sossio Serra, Giovanni de Simone

**Affiliations:** 1 Hypertension Research Center, Federico II University Hospital of Naples, Via Pansini 5, bld #1, 80131 Naples, Italy; 2 Department of Advanced Biomedical Science, Federico II University Hospital, Naples, Italy; 3 Department of Traslational Medical Science. Federico II University Hospital, Naples, Italy; 4 Emergency Department, Bufalini Hospital, Cesena, Italy

**Keywords:** Acute dyspnea, Multi-organ ultrasound, Pocket-sized device, Emergency department

## Abstract

**Background:**

The availability of ultra-miniaturized pocket ultrasound devices (PUD) adds diagnostic power to the clinical examination. Information on accuracy of ultrasound with handheld units in immediate differential diagnosis in emergency department (ED) is poor. The aim of this study is to test the usefulness and accuracy of lung ultrasound (LUS) alone or combined with ultrasound of the heart and inferior vena cava (IVC) using a PUD for the differential diagnosis of acute dyspnea (AD).

**Methods:**

We included 68 patients presenting to the ED of “Maurizio Bufalini” Hospital in Cesena (Italy) for AD. All patients underwent integrated ultrasound examination (IUE) of lung-heart-IVC, using PUD. The series was divided into patients with dyspnea of cardiac or non-cardiac origin. We used 2 × 2 contingency tables to analyze sensitivity, specificity, positive predictive value and negative predictive value of the three ultrasonic methods and their various combinations for the diagnosis of cardiogenic dyspnea (CD), comparing with the final diagnosis made by an independent emergency physician.

**Results:**

LUS alone exhibited a good sensitivity (92.6%) and specificity (80.5%). The highest accuracy (90%) for the diagnosis of CD was obtained with the combination of LUS and one of the other two methods (heart or IVC).

**Conclusions:**

The IUE with PUD is a useful extension of the clinical examination, can be readily available at the bedside or in ambulance, requires few minutes and has a reliable diagnostic discriminant ability in the setting of AD.

## Background

Acute dyspnea is one of the most frequent symptoms of patients presenting to the emergency department (ED), with about 4–5 million hits per year in the United States [1]. The differential diagnosis is often challenging especially to distinguish between dyspnea of cardiac origin (CD) and dyspnea of other causes. Medical history, physical examination, blood gas analysis, electrocardiogram, laboratory tests and chest X-rays are essential in the diagnostic process, but sometimes not enough and often difficult to obtain instantaneously. For these reasons, about 20% of patients who logs in ED for dyspnea receive incorrect diagnosis and consequently inadequate therapy [2].

Focused cardiac ultrasound plays an important role in the diagnostic evaluation of the patient at bedside and it is important for triage decisions and emergency treatment [3]. Large amount of information can be also obtained using lung ultrasound (LUS) especially in the identification and evaluation of pleuro-pulmonary diseases and in the evaluation of extra-vascular lung water [4, 5]. Utility of LUS has been recently tested to help discriminating causes of acute dyspnea in ED, with or without simultaneous evaluation of heart and inferior vena cava (IVC) [6, 7]. Using pocket-size imaging device also the assessment of extravascular lung water with evaluation of B-lines and pleural effusion is feasible and reliable [8].

In the setting of ED standard echocardiographic equipment may be heavy and difficult to handle while hand-carried ultrasound devices have been developed for bedside use. In particular, the ultrasound technology pool has been enriched with pocket ultrasound devices that offer advantages in terms of portability and speed, and are able to reproduce images with standard ultrasound and color Doppler. These devices can be used as first ultrasound approach in ED and ambulance and confer added diagnostic power to the clinical examination in populations of patients with no history of cardiovascular disease [9, 10].

Informations on accuracy of first diagnostic assessment with pocket ultrasound device in immediate differential diagnosis for acute dyspnea in ED are still deficient. The aim of this study is to test the utility and accuracy of LUS alone or combined with ultrasound of the heart and IVC in the identification of CD with pocket ultrasound device in ED.

## Methods

**Fig. 1:**
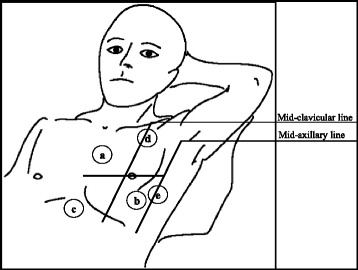
Integrated ultrasound examination. **a**, **b**, **c**: heart and IVC examination. **d**, **e** lung ultrasound examination. **a**: parasternal long/short axis viex; **b**: apical view; **c**: subcostal view; **d**: anteriorly on the II intercostal space, midclavicular line; **e**: V intercostal space, midaxillary line

From November 2014 to August 2015 we enrolled 68 patients presenting to the ED of “Maurizio Bufalini” hospital in Cesena (Italy) for acute dyspnea or sudden worsening of chronic dyspnea within the previous 48 h. All patients underwent clinical exam, blood gas analysis, chest X-Ray, ECG, routine blood tests and integrated ultrasound examination of lung-heart-IVC with pocket size device Vscan (General Electric Healthcare) with a single probe (1.7–3.8 MHz), using abdominal preset for lung and cardiac preset for heart and IVC. Ultrasound examination was performed in semi sitting or supine position by one emergency physician expert in transthoracic echocardiography (ASE level III) and with good experience of LUS who was not taking care of the patient (Fig. 1). The ultrasound examination was done within 30 min from the arrival of the patients in ED.

Thorax was examined following a simplified protocol that provides two scans at each side: anteriorly on the II intercostal space, midclavicular line and lateral on the V intercostal space, midaxillary line, to sample upper and lower lungs [5, 11]. The presence or the absence of interstitial syndrome (IS, defined as the presence of at least 3 B-lines for field) and the presence or the absence of pleural effusion (defined as a hypo-anechoic space between the parietal and visceral pleura) were evaluated. LUS was defined positive for bilateral IS and/or effusion if any IS and/or effusion was present in at least 1 scan per side and symmetrically [12]. By symmetrical we mean the presence of IS and/or effusion in the same scans in both sides of the lungs.

The heart was examined in at least one projection (parasternal long/short axis view and/or apical view and/or subcostal view) allowing qualitative evaluation of left ventricular systolic function, size of chambers and the presence or absence of pericardial effusion. Ejection fraction (EF) was estimated visually and categorized as preserved if >40% or reduced if ≤40% [13].

The IVC was explored in subcostal view and evaluated by the presence or absence of dilatation (> 2 cm) and hypo-reactivity with breathing (variation of size <50%) [3].

The final diagnosis of acute dyspnea was determined by an independent emergency physician (blind to the integrated ultrasound examination) who has followed each patient, taking into account all the clinical investigations performed (clinical exam, blood gas analysis, chest X-Ray, ECG, routine blood tests) and the evolution of the patients (response to diuretics, vasoactive agents, non-invasive ventilation, corticosteroids, etc.). Without knowledge of the ultrasound data collected in the ED, he had to classify patients into 2 groups: cardiogenic dyspnea (CD) and dyspnea of non-cardiac origin (non-CD). The primary end point was to compare the diagnostic performance of cardiopulmonary ultrasound with pocket size device and standard examinations (clinical, laboratory, chest x-ray, ECG) for the diagnosis of CD. Specifically, for the diagnosis of CD, the Boston-score diagnostic criteria (points 5–12) have been always satisfied [14].

In patients with coexistence of heart failure and another cause of dyspnea the main diagnosis was considered CD [15].

Another emergency physician expert in lung and cardiac ultrasound read all images, blind to the final diagnosis from the ED.

### Statistics

Data were analyzed using SPSS version 21.0 (SPSS, Chicago, Illinois, USA). Continuous data are expressed as mean ± 1 standard deviation and categorical variables as percentages. Quantitative variables were compared by using Student’s *t*-test while χ ^2^ distribution was used to compare categorical variables.

The population was divided into patients with CD and patients with non-CD. Contingency tables were produced to analyze sensitivity, specificity, positive predictive value and negative predictive value of the three ultrasonic findings obtained with pocket size device and their various combinations for the diagnosis of CD, based on the final diagnosis made by the emergency physician. Receiver operating characteristic (ROC) curves (AUC) are used to describe and compare the performance of each different ultrasound modality (Lung, Heart, IVC) and the combination between bilateral IS/effusion and only 1 of the cardiovascular abnormalities (EF ≤40% OR dilated and not collapsing IVC) versus the final ED diagnosis [16].

A *p*-value <0.05 was considered statistical significant.

## Results

Our study population included 68 patients (43 males) with a mean age of 78 years. Table 1 shows the baseline characteristics of the population at arrival in ED. An integrated ultrasound examination was feasible in all patients except 4 who were excluded from the IVC evaluation because under CPAP at arrival in ED. The ultrasound examination time was always less than 3 min.

**Table 1:** Baseline characteristics and in-hospital arrival vital signs of the study population

Characteristics	Total study population (*n* = 68)	Dyspnea of cardiac origin (*n* = 27)	Dyspnea of non- cardiac origin (*n* = 41)
Age (years)	78 ± 12	80 ± 9	77 ± 14
Men (%)	62	56	66
Heart Rate (bpm)	97 ± 25	91 ± 27	100 ± 24
Systolic BP (mmHg)	135 ± 24	138.7 ± 26.4	132.9 ± 22.6
Diastolic BP (mmHg)	78 ± 15	81.8 ± 14.9	75.3 ± 14.3
Respiratory rate (breaths/min)	24 ± 6	22 ± 6	25 ± 6
Oxygen saturation (%)	92 ± 6	93 ± 4	92 ± 7
PaO_2_/Fio_2_	269 ± 92	292 ± 75	254 ± 100
Body temperature (°C)	37 ± 1	36 ± 1*	37 ± 1
White Blood cell (×10^3^/ml)	12.0 ± 5.8	10.4 ± 5.5	13.1 ± 5.7
Neutrophils (%)	77 ± 11	73 ± 10*	79 ± 11
Hemoglobin (g/dl)	12.7 ± 2.3	12.3 ± 2.1	12.9 ± 2.4
C reactive protein (mg/dl)	66 ± 79	51 ± 71	73 ± 82
Lactate (mmol/L)	2.4 ± 2.1	2.6 ± 2.9	2.3 ± 1.3
Creatinine (mg/dl)	1.2 ± 0.9	1.48 ± 1.21	1.09 ± 0.47
History of Chronic Obstructive Pulmonary disease (%)	49	32*	59
History of Heart Failure (%)	41	60*	29

*BP* Blood Pressure

*p > 0.05 vs dyspnea of non-cardiac origin

Table 2 displays the definitive diagnosis of patients, 40% of whom had CD, while 60% had non-CD. Patients with CD show lower body temperature and percentage of neutrophils on white blood cell count (both *p* < 0.05) and have tendencies to have higher Lactate and creatinine levels and lower C-reactive protein compared to patients with non-CD (Table 1).

**Table 2:** Final diagnosis at ED discharge

	Cases
Final diagnosis	
Acute heart failure	25 (36.8%)
Pneumonia	18 (26.5%)
Acute or re-exacerbation of COPD	17 (25%)
Acute heart failure + Pneumonia	2 (2.9%)
Others: (3) lung cancer, (2) pulmonary embolism, (1) pneumothorax	6 (8.8%)
Identification of dyspnea of cardiac origin	
Dyspnea of cardiac origin	27 (39.7%)
Dyspnea of non-cardiac origin	41 (60.3%)

*COPD* Chronic Obstructive Pulmonary Disease

Table 3 shows the sensitivity, specificity, positive predictive value (PPV), negative predictive value (NPV) and accuracy for the diagnosis of CD of various ultrasound markers taken individually or in combination. LUS positivity for bilateral IS and/or effusion exhibited good sensitivity and good specificity. The maximum accuracy (90%) for the diagnosis of CD was obtained in the combination of LUS positivity for bilateral IS and/or effusion AND reduced EF OR dilated and hypo-reactive IVC, while the contemporary presence of all three ultrasound abnormalities did not give an optimal accuracy, especially because of the poor sensitivity.

**Table 3:** Sensitivity, specificity, positive and negative predictive value and accuracy of the Chest X-ray, Boston score (points 8–12), lung, heart and IVC ultrasound and their combinations in the diagnosis of dyspnea of cardiac origin

Parameter	Sensitivity (%)	Specificity (%)	PPV (%)	NPV (%)	Accuracy (%)
Chest X-ray	75 (52.9–89.3)	85.4 (70.1–93.9)	75 (52.9–89.4)	85.4 (70.1–93.9)	82
Boston score (points 8–12)	79.2 (57.3–92)	70.7 (54.3–83.3)	61.3 (42.3–77.6)	85.3 (68.2–94.4)	74
IS/ effusion	92,6 (74.2–98.7)	80,5 (64.6–90.6)	75,8 (57.4–88.2)	94,3 (79.5–99)	85,3
IVC dilated and not collapsing	65,4 (44.3–82)	88,2 (71.6–96.2)	81 (57.4–93.7)	76,9 (60.2–88.2)	78,3
EF ≤ 40%	66,7 (46–82.7)	87,2 (71.7–95.2)	78,3 (55.8–91.7)	79,1 (63.5–89.4)	78,8
IS/effusion OR IVC dilated and not collapsing	100 (84.4–100)	71.4 (53.4–84.7)	72.9 (55.6–85.6)	100 (83.4–100)	85
IS/effusion OR EF ≤ 40%	92,3 (74.2–98.7)	70,7 (54.3–83.3)	67.5 (50.1–81.4)	93.5 (77.2–98.9)	80
IS/effusion AND IVC dilated and not collapsing	57,7 (37.2–76)	94,1 (78.9–98.9)	88,2 (62.2–97.9)	74,4 (58.6–86)	78,3
IS/effusion AND EF ≤ 40%	66.6 (46–82.7)	97,5 (85.5–99.8)	94,7 (71.8–99.7)	81.6 (71.8–99.7)	85
IS/effusion AND EF ≤ ≤40% AND IVC dilated and not collapsing	40.7 (23–60.9)	97,6 (85.6–99.8)	91,6 (59.7–99.5)	71.4 (57.6–82.3)	73,3
IS/effusion AND (EF ≤ 40% OR IVC dilated and not collapsing)	81.4 (61.2–92.3)	95.1 (82.2–99.2)	91.7 (71.6–98.6)	88.6 (74.6–95.7)	90

*PPV* Positive Predictive Value, *NPV* Negative Predictive Value, *IS* Bilateral and symmetrical Interstitial Syndrome, *IVC* Inferior Vena Cava, *EF* Ejection Fraction

**Fig. 2:**
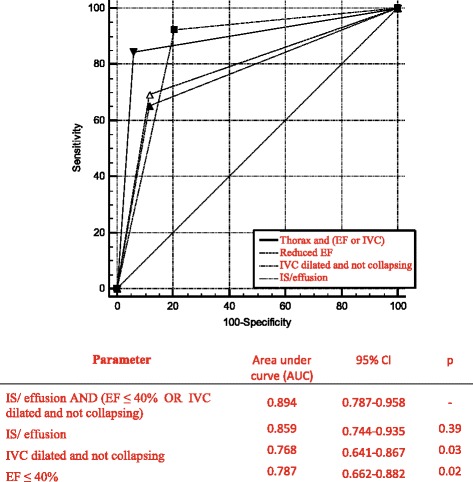
Receiver operating characteristic (ROC) curve comparing accuracy of different ultrasound modalities. p vs IS/effusion AND EF ≤ 40% OR IVC dilated and not collapsing

Figure 2 shows receiving operating characteristics (ROC) curves (AUC) of the different ultrasound diagnostic approaches evaluated. The combined presence of bilateral IS/effusion AND only 1 other cardiovascular abnormalities (EF ≤40% OR dilated and not collapsing IVC) has the highest AUC for the identification of patients with dyspnea of cardiac origin. Although the difference is not statistically significant from the LUS alone, specificity is maximized.

## Discussion

In a population of patients presenting to the ED for the recent onset of acute dyspnea the integrated ultrasound examination of Lung-Heart-IVC with a pocket size device is feasible and has reliable diagnostic value for the diagnosis of dyspnea of cardiac origin.

Among clinical manifestations of cardiovascular disease, heart failure is increasing in prevalence and incidence, and is among the leading causes of hospitalization and death. Despite advances in recognize subclinical findings of impaired left ventricular function, in acute heart failure the diagnosis is challenging [17–19]. Diastolic dysfunction of the left ventricle, present in all the types of heart failure, regardless of the values of EF, is characterized by increased left ventricular filling pressure with congestion or pulmonary edema and consequent respiratory symptoms. The dysfunction of the right heart, usually secondary to increase in pulmonary arterial pressure, is responsible for the appearance of the signs of peripheral congestion in heart failure (peripheral edema, liver stasis and distended jugular veins).

Even in the ED diagnosis of acute heart failure is mostly based on clinical examination. However, signs and symptoms of acute heart failure are not specific. Even chest x-ray may be falsely negative in 20% of cases [20]. The Boston score that we adopted has a validated prognostic value in Italy and has proved to be more accurate in predicting poor prognosis than other criteria [21].

In our study the diagnostic accuracy of chest X-ray and Boston clinical criteria risk score ≥ 8 was suboptimal for detection of patients with acute heart failure and was much lower compared to integrated ultrasound examination of Lung-Heart-IVC. As already demonstrated, LUS performs better than Chest X-ray in identification of patients with acute heart failure, probably because of poor quality of X-ray in the setting of ED [15, 20].

LUS can be of great help in differentiating CD from non-CD, especially in the case of borderline Boston score (between 5 and 7). The presence of numerous B-lines, sometimes confluent, configures the framework of an alveolar-interstitial syndrome [22]. It may reflect the presence of either cardiogenic or inflammatory edema, or fibrosis [12]. Pleural effusion may instead be the result of inflammation of the lung parenchyma or heart failure with increased central venous pressure [23].

In our analysis, the presence of bilateral IS and/or effusion exhibits a high sensitivity for the diagnosis of CD, with a high negative predictive value, confirming the pathophysiological assumption that in acute heart failure interstitial edema or effusion are present bilaterally and confirming the diagnostic importance of LUS in patients with decompensated heart failure [24, 25]. Among patients with heart failure in whom bilateral IS is absent, right heart dysfunction might be prevalent. In addition, specificity and, especially, positive predictive value are suboptimal, probably due to presence of lung IS also in the case of pulmonary fibrosis, acute respiratory distress syndrome, interstitial pneumonia, bilateral pneumonia. Although there are ultrasound signs to help differentiating cardiogenic pulmonary edema from ARDS, these are not easily recognizable, especially in an emergency setting with portable devices with cardiac probe [26]. One limitation of our approach in LUS should be highlighted: we decided to adopt a simplified protocol with only four zone scanning protocol to speed up the echo examination time. In the setting of ED this has already been done using a simplified six zone scanning protocol obtaining a sensitivity for LUS alone comparable to what we have found [23]. A more comprehensive scanning would have been optimal but also time consuming and so not feasible facing patients in critical condition.

The presence of EF less than or equal to 40% has a low sensitivity for the diagnosis of dyspnea related to acute cardiac decompensation. This reflects the fact that a significant proportion of patients with HF presents with preserved EF [27]. However, as expected, the specificity is good.

The presence of dilated and not collapsing IVC is typically an accurate index of increased right atrial pressure that can be a result of either an increased pulmonary arterial pressure or right ventricular dysfunction with or without significant tricuspid regurgitation [28]. In our study, this index exhibits low sensitivity for the diagnosis of cardiogenic dyspnea. This is probably because a sudden increase in pulmonary capillary wedge pressure has no immediate effect on the pulmonary arterial circulation. However, the combination of dilation and hypo-reactivity of the IVC carries good specificity for the diagnosis of cardiogenic dyspnea with an acceptable positive predictive value. Thus we partially confirm the finding that a dilated and not collapsing IVC has acceptable correlation with increased left ventricular pressure [29], though IVC can be dilated and not collapsing also in different acute (pulmonary embolism, pneumonia with severe hypoxemia) or chronic (pulmonary hypertension in chronic heart failure, chronic pulmonary heart disease) conditions as well as in myocardial infarction with right ventricular dysfunction [28].

The combination of bilateral IS/effusion and EF ≤40% has a better accuracy than the association between bilateral IS/effusion and dilated and not collapsing IVC for the diagnosis of CD. The contemporary presence of all three ultrasound abnormalities has not an optimal accuracy, especially because of the poor sensitivity.

In the diagnosis of acute heart failure the combination of reduced left ventricular EF and poor collapsibility of IVC has been demonstrated to have high specificity and sensitivity for the diagnosis of HF [30], with an adjunctive specificity value for the LUS. In our paper we expand this finding demonstrating that a combination of positive LUS examination and reduced EF or IVC dilated and not collapsing demonstrates the best accuracy in the diagnosis of dyspnea of cardiac origin, mainly due to the ability of this combination to maximize specificity compare to LUS alone.

Consistently, the association between bilateral IS/effusion and only 1 of the cardiovascular abnormalities (EF ≤40% or dilated and not collapsing IVC) improves the sensitivity and accuracy compared to the association between bilateral IS/effusion and EF ≤ 40%, because it captures also patients with HF with preserved ejection fraction among whom many have dilated and not collapsing IVC.

The diagnostic value of lung-cardiac-IVC integrated ultrasound was recently tested by *Kajimoto* et al. who showed accuracy (93.3%) slightly greater than our study. This difference possibly reflects the fact that in our protocol color Doppler was not available and only EF was the parameter of LV function detectable during cardiac ultrasound, whereas in their protocol cardiac ultrasound was considered diagnostic also with EF >40% when associated with moderate to severe mitral regurgitation [7] expanding the spectrum of types of recognizable heart failure.

### Limitations

Our study has some limitations. First, the population sample could be larger. However our findings are consistent with previous reports [7, 12]. It was not possible to follow the clinical course of all enrolled patients because 7 of them were admitted to a different hospital after the ED evaluation. In the remaining 61 patients the EP diagnosis has been confirmed in 93.4% of the cases at the final hospital discharge. Since our aim was to evaluate the diagnostic accuracy of ultrasound in the ED we decided to adopt as diagnostic gold standard the EP diagnosis. Also it is not yet possible with the pocket ultrasound device to examine the diastolic function of the left ventricle.

## Conclusions

Overall, the integrated lung-heart-IVC ultrasound examination improves the accuracy of LUS alone, by maximizing specificity, and allowing to capture different types of heart failure. This makes pocket ultrasound devices useful for the efficiency and speed in the differential diagnosis of acute dyspnea in the ED.

The IUE of lung-heart-inferior vena cava with pocket ultrasound devices is an extension of the clinical examination and can be realized with a protocol that provides 4 thoracic scans (2 front and 2 lateral), at least one view (parasternal and/or apical and/or subcostal) which enables the assessment of LV systolic function and the subcostal view for the inferior vena cava. This method is readily available at bedside or even in ambulance, requires few minutes and has reliable diagnostic accuracy in the management of acute dyspnea.

## Abbreviations

AD: Acute dyspnea
CD: Cardiac dyspnea
ED: Emergency department
EF: Ejection fraction
HF: Heart failure
IS: Interstitial syndrome
IUE: Integrated ultrasound examination
IVC: Inferior vena cava
LUS: Lung ultrasound
NPV: Negative predictive value
PPV: Positive predictive value
PUD: Pocket size device

## References

[1] Wang CS, FitzGerald JM, Schulzer M, Mak E, Ayas NT (2005). Does this dyspneic patient in the emergency department have congestive heart failure?. JAMA.

[2] Ray P, Birolleau S, Lefort Y, Becquemin MH, Beigelman C, Isnard R, et al. Acute respiratory failure in the elderly: etiology, emergency diagnosis and prognosis. Crit Care. 2006;10:R82. 10.1186/cc4926 PMC1550946 16723034

[3] Labovitz AJ, Noble VE, Bierig M, Goldstein SA, Jones R, Kort S, et al. Focused cardiac ultrasound in the emergent setting: a consensus statement of the American Society of Echocardiography and American College of Emergency Physicians. J Am Soc Echocardiogr. 2010;23(12):1225–30. 10.1016/j.echo.2010.10.005 21111923

[4] Copetti R, Soldati G. (2012). Ecografia Toracica seconda edizione. C.G. Edizioni Medico Scientifiche.

[5] Picano E, Pellikka PA (2016). Ultrasound of extravascular lung water: a new standard for pulmonary congestion. Eur Heart J.

[6] Cibinel GA, Casoli G, Elia F, Padoan M, Pivetta E, Lupia E, et al. Diagnostic accuracy and reproducibility of pleural and lung ultrasound in discriminating cardiogenic causes of acute dyspnea in the Emergency Department. Intern Emerg Med. 2012;7:65. 10.1007/s11739-011-0709-1 22033792

[7] Kajimoto K, Madeen K, Nakayama T, Tsudo H, Kuroda T, Abe T (2012). Rapid evaluation by lung-cardiac-inferior vena cava (LCI) integrated ultrasound for differentiating heart failure from pulmonary disease as the cause of acute dyspnea in the emergency setting. Cardiovasc Ultrasound.

[8] Sicari R, Galderisi M, Voigt JU, Habib G, Zamorano JL, Lancellotti P, et al. The use of pocket-size imaging devices: a position statement of the European Association of Echocardiography. Eur J Echocardiogr. 2011;12(2):85–7. 10.1093/ejechocard/jeq184 21216764

[9] Galderisi M, Santoro A, Versiero M, Schiano Lomoriello V, Esposito R, Raia R, et al. Improved cardiovascular diagnostic accuracy by pocket size imaging device in non-cardiologic outpatients: the NaUSiCa (Naples Ultrasound Stethoscope in Cardiology) study. Cardiovasc Ultrasound. 2010;8:51. 10.1186/1476-7120-8-51 PMC3003628 21110840

[10] Testuz A, Müller H, Keller PF, Meyer P, Stampfli T, Sekoranja L, et al. Diagnostic accuracy of pocket-size handheld echocardiographs used by cardiologists in the acute care setting. Eur Heart J Cardiovasc Imaging. 2013;14(1):38–42. 10.1093/ehjci/jes085 22535657

[11] Volpicelli G, Elbarbary M, Blaivas M, Lichtenstein DA, Mathis G, Kirkpatrick AW, et al. International evidence-based recommendations for point-of-care lung ultrasound. Intensive Care Med. 2012;38(4):577–91. 10.1007/s00134-012-2513-4 22392031

[12] Gargani L (2011). Lung ultrasound: a new tool for the cardiologist. Cardiovasc Ultrasound.

[13] Kimura BJ, Yogo N, O'Connell CW, Phan JN, Showalter BK, Wolfson T (2011). Cardiopulmonary limited ultrasound examination for “quick-look” bedside application. Am J Cardiol.

[14] Remes J, Miettinen H, Reunanen A, Pyorala K (1991). Validity of clinical diagnosis of heart failure in primary health care. Eur Heart J.

[15] Pirozzi C, Numis FG, Pagano A, Melillo P, Copetti R, Schiraldi F (2014). Immediate versus delayed integrated point-of-care-ultrasonography to manage acute dyspnea in the emergency department. Crit Ultrasound J.

[16] Hanley JA, McNeil BJ (1983). A method of comparing the areas under receiver operating characteristic curves derived from the same cases. Radiology.

[17] Mancusi C, Gerdts E, de Simone G, Midtbø H, Lønnebakken MT, Boman K, et al. Higher pulse pressure/stroke volume index is associated with impaired outcome in hypertensive patients with left ventricular hypertrophy the LIFE study. Blood Press. 2017;26(3):150–5. 10.1080/08037051.2016.1243009 27710139

[18] De Marco M, Gerdts E, Mancusi C, Roman MJ, Lønnebakken MT, Lee ET, et al. Influence of left ventricular stroke volume on incident heart failure in a population with preserved ejection fraction (from the strong heart study). Am J Cardiol. 2017;119(7):1047–52. 10.1016/j.amjcard.2016.12.011 PMC5348268 28159195

[19] de Simone G, Izzo R, Losi MA, Stabile E, Rozza F, Canciello G, Mancusi C, Trimarco V, De Luca N, Trimarco B (2016). Depressed myocardial energetic efficiency is associated with increased cardiovascular risk in hypertensive left ventricular hypertrophy. J Hypertens.

[20] Collins SP, Lindsell CJ, Storrow AB, Abraham WT (2006). Prevalence of negative chest radiography results in the emergency department patient with decompensated heart failure. Ann Emerg Med.

[21] Di Bari M, Pozzi C, Cavallini MC, Innocenti F, Baldereschi G, De Alfieri W, et al. The diagnosis of heart failure in the community. J Am Coll Cardiol. 2004;44:1601. 10.1016/j.jacc.2004.07.022 15489092

[22] Lichtenstein D, Mézière G, Biderman P, Gepner A, Barré O (1997). The comet-tail artifact: an ultrasound sign of alveolar-interstitial syndrome. Am J Respir Crit Care Med.

[23] Natanzon A, Kronzon I (2009). Pericardial and pleural effusions in congestive heart failure-anatomical, pathophysiologic, and clinical considerations. Am J Med Sci.

[24] Pivetta E, Goffi A, Lupia E, Tizzani M, Porrino G, Ferreri E, et al. Lung ultrasound-implemented diagnosis of acute decompensated heart failure in the ED: a SIMEU multicenter study. Chest. 2015;148(1):202–10. 10.1378/chest.14-2608 25654562

[25] Miglioranza MH, Gargani L, Sant'Anna RT, Rover MM, Martins VM, Mantovani A, et al. Lung ultrasound for the evaluation of pulmonary congestion in outpatients: a comparison with clinical assessment, natriuretic peptides, and echocardiography. JACC Cardiovasc Imaging. 2013;6(11):1141–51. 10.1016/j.jcmg.2013.08.004 24094830

[26] Copetti R, Soldati G, Copetti P (2008). Chest sonography: a useful tool to differentiate acute cardiogenic pulmonary edema from acute respiratory distress syndrome. Cardiovasc Ultrasound.

[27] Nieminen MS, Brutsaert D, Dickstein K, Drexler H, Follath F, Harjola VP, et al. EuroHeart Failure Survey II (EHFS II): a survey on hospitalized acute heart failure patients: description of population. Eur Heart J. 2006;27(22):2725–36. 10.1093/eurheartj/ehl193 17000631

[28] Brennan JM, Blair JE, Goonewardena S, Ronan A, Shah D, Vasaiwala S, et al. Reappraisal of the use of inferior vena cava for estimating right atrial pressure. J Am Soc Echocardiogr. 2007;20:857. 10.1016/j.echo.2007.01.005 17617312

[29] Blair JE, Brennan JM, Goonewardena SN, Shah D, Vasaiwala S, Spencer KT (2009). Usefulness of hand-carried ultrasound to predict elevated left ventricular filling pressure. Am J Cardiol.

[30] Anderson KL, Jenq KY, Fields JM, Panebianco NL, Dean AJ (2013). Diagnosing heart failure among acutely dyspneic patients with cardiac, inferior vena cava, and lung ultrasonography. Am J Emerg Med.

